# Computational discovery of co-expressed antigens as dual targeting candidates for cancer therapy through bulk, single-cell, and spatial transcriptomics

**DOI:** 10.1093/bioadv/vbae096

**Published:** 2024-06-20

**Authors:** Evgenii Chekalin, Shreya Paithankar, Rama Shankar, Jing Xing, Wenfeng Xu, Bin Chen

**Affiliations:** Department of Pediatrics and Human Development, Michigan State University, Grand Rapids, MI 49503, United States; Department of Pediatrics and Human Development, Michigan State University, Grand Rapids, MI 49503, United States; Department of Pediatrics and Human Development, Michigan State University, Grand Rapids, MI 49503, United States; Department of Pediatrics and Human Development, Michigan State University, Grand Rapids, MI 49503, United States; Hengenix Biotech, Inc., Milpitas, CA 95035, United States; Department of Pediatrics and Human Development, Michigan State University, Grand Rapids, MI 49503, United States; Department of Pharmacology and Toxicology, Michigan State University, East Lansing, MI 48824, United States; Department of Computer Science and Engineering, Michigan State University, East Lansing, MI 48824, United States

## Abstract

**Motivation:**

Bispecific antibodies (bsAbs) that bind to two distinct surface antigens on cancer cells are emerging as an appealing therapeutic strategy in cancer immunotherapy. However, considering the vast number of surface proteins, experimental identification of potential antigen pairs that are selectively expressed in cancer cells and not in normal cells is both costly and time-consuming. Recent studies have utilized large bulk RNA-seq databases to propose bispecific targets for various cancers. However, co-expressed pairs derived from bulk RNA-seq do not necessarily indicate true co-expression of both markers in malignant cells. Single-cell RNA-seq (scRNA-seq) can circumvent this issue but the issues in low coverage of transcripts impede the large-scale characterization of co-expressed pairs.

**Results:**

We present a computational pipeline for bsAbs target identification which combines the advantages of bulk and scRNA-seq while minimizing the issues associated with using these approaches separately. We select hepatocellular carcinoma (HCC) as a case study to demonstrate the utility of the approach. First, using the bulk RNA-seq samples in the OCTAD database, we identified target pairs that most distinctly differentiate tumor cases from healthy controls. Next, we confirmed our findings on the scRNA-seq database comprising 39 361 healthy cells from vital organs and 18 000 cells from HCC tumors. The top pair was GPC3–MUC13, where both genes are co-expressed on the surface of over 30% of malignant hepatocytes and have very low expression in other cells. Finally, we leveraged the emerging spatial transcriptomic to validate the co-expressed pair *in situ*.

**Availability and implementation:**

A standalone R package (https://github.com/Bin-Chen-Lab/bsAbsFinder).

## 1 Introduction

Bispecific antibodies (bsAbs) are man-made antibodies which simultaneously bind to two different antigens. The term ‘bispecific antibody’ was first coined over 50 years ago by Nisonoff and colleagues (Nisonoff *et al.* 1960; Labrijn *et al.* 2019). More than 200 bsAbs are currently in clinical development and nine bsAbs have been approved for cancer therapy in the past three years (2021–2023), suggesting the immense interest of bsAbs in drug discovery (Klein *et al.* 2024).

bsAbs come in various formats, each designed to address specific needs in production, stability, and therapeutic efficacy. These formats include fragment-based bsAbs, which combine antigen-binding fragments without an Fc region, symmetric bsAbs that incorporate both specificities in a single polypeptide chain, and asymmetric bsAbs that retain the native antibody architecture to closely resemble the natural antibodies and maintain the inherent functional traits (Labrijn *et al.* 2019).

bsAbs offer novel therapeutic opportunities by enabling the targeting of multiple pathways simultaneously, facilitating immune cell engagement, and overcoming resistance mechanisms. Their versatility and diverse formats make them valuable tools in the development of innovative therapies for not only cancer but also non-cancer diseases. For instance, emicizumab facilitates factor X activation by binding to factors IXa and X simultaneously, mimicking the action of factor VIII. This mechanism offers a novel approach to hemophilia A treatment by enhancing blood clotting in patients with impaired or absent factor VIII expression (Labrijn *et al.* 2019).

For cancer immunotherapy, various types of bsAbs have been explored based on the types of biological targets and modes of action. The majority of bsAbs under clinical investigation are bispecific immune cell engagers where one arm of the bsAbs targets an established immune cell antigen and another arm links to a tumor cell antigen. For instance, blinatumomab targets the CD3 antigen of T-cells and CD19 surface antigen of B cells for the treatment of B-cell malignancies (Huang *et al.* 2020). Another less-studied bsAbs class aims to target two tumor-associated antigens such that the drug could improve selectivity toward tumor cells, while minimizing side effects in normal tissues, or modulate two functional pathways in the tumor to overcome treatment resistance (Sellmann *et al.* 2016, Huang *et al.* 2020). Examples include amivantamab, targeting EGFR and MET for non-small cell lung cancer; petosemtamab, targeting EGFR and LGR5 for head and neck squamous cell carcinoma; and izalontamab, which targets EGFR and HER3 for locally advanced or metastatic epithelial tumors (Klein *et al.* 2024). Similarly, chimeric antigen receptor (CAR) T-cell that simultaneously target two tumor-associated antigens can also enhance antitumor activity and circumvent escape mechanisms in solid tumors (Labrijn *et al.* 2019). Recent advances in protein engineering allow the creation of proteins tailored to target specific cells using combinations of surface antigens (Hirabayashi *et al.* 2021, Spiegel et al. 2021).

However, despite these advancements, most possible combinations of antigens for targeting various cells have not been thoroughly investigated. Considering the wide range of surface antigens yielding millions of possible bsAbs pairs to evaluate, computational identification of bsAbs target pairs that are expressed only in cancer cells but not in normal cells has promising application in bsAbs-based cancer immunotherapy.

As rich bulk RNA-seq data has been generated for various cancers and normal tissues, it has been extensively reused to pinpoint therapeutic targets and biomarkers. A recently proposed computational approach employed Boolean logic to characterize highly co-expressed antigen pairs in tumor versus healthy samples in bulk RNA-seq databases. They utilized large bulk RNA-seq databases such as The Cancer Genome Atlas Program (TCGA, https://www.cancer.gov/tcga) and Genotype-Tissue Expression portal (GTEx) as sources of tumors and healthy control to identify novel CAR T-cells marker candidates (Dannenfelser *et al.* 2020). However, heterogenous cell populations of bulk RNA-seq limit its potential to discern the precise expression of targets at the individual cell level within cancer tissues, potentially leading to a significant number of false-positive antigen pairs. Whereas, scRNA-seq offers a high-resolution quantification of target expression in individual cells, but the challenges in accurate identification of cell types and the low coverage of transcripts restrict its direct application in bsAbs discovery.

**Figure 1.**
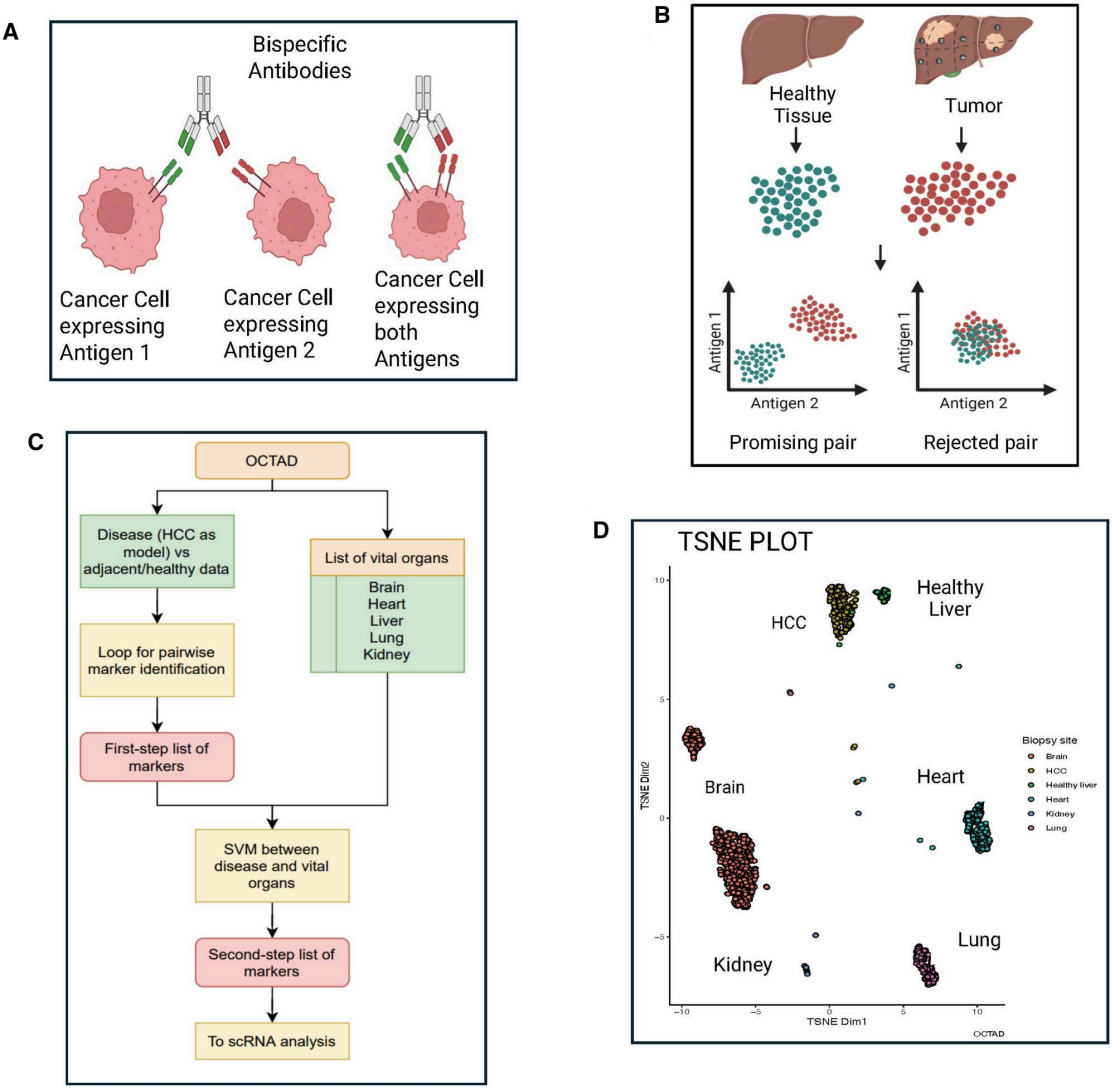
Identification of bulk RNA-seq-based bsAbs pairs. (A) bispecific antibody mechanism, (B) schematic of the bulk RNA-seq based bsAbs pair selection, (C) workflow of bsAbs pair selection, and (D) *t*-distributed stochastic neighbor embedding (*t*-SNE) plot of the OCTAD data for HCC, normal liver, and normal vital organs used for the verification of the non-specific expression of the proposed bsAbs target pairs.

bsAbs can target malignant cells expressing both antigens thus enhancing therapeutic efficacy and selectivity. Alternately, they can target distinct malignant cell subpopulations where either antigen is expressed, widening therapeutic applicability in heterogenous tumors (Fig. 1A).

In this study, we introduce a computational pipeline for identifying bsAbs targets that leverages the strengths of bulk and scRNA-seq while mitigating the challenges typically associated with using these methods individually. We first harnessed high-quality bulk RNA-seq data from OCTAD database (compiled from multiple open databases) to identify novel pairs. We developed computational approaches to find the pairs with a similar high expression level within tumor samples (case) and a low or zero expression level within non-tumor samples (control) (Fig. 1B). Along with normal and/or adjacent normal tissues related to the given cancer, normal vital organs were also included in the control dataset. The resulting pairs from bulk RNA-seq analysis were subsequently verified in an assembled scRNA-seq dataset to confirm their co-expression in the malignant cell type (Fig. 1C). Further, spatial transcriptomic data were used as external validation to visualize the co-expression pattern of the top pair in cancerous and healthy tissues. The utility of the proposed pipeline was demonstrated in hepatocellular carcinoma (HCC) bsAbs discovery, and similar code could be applied to identify bsAbs targets for other cancers including prostate cancer shown as another case study in the code.

## 2 Methods

### 2.1 Data collection and preprocessing

#### 2.1.1 Bulk RNA-seq database

We utilized the OCTAD database, a collection of databases including TCGA, GTEx, and MET500 (Robinson *et al.* 2017). It contains a total of 19 127 samples including 11 715 samples from 130 distinct cancers and 1950 healthy samples from five vital organs (1148 brain samples from various regions, 376 heart samples from either the left ventricle or atrial appendage, 28 samples from the kidney cortex, 110 liver samples, and 288 lung samples) that were processed from raw sequence data under the same pipeline (Zeng *et al.* 2019). Recent studies demonstrated the feasibility of performing integrative analysis of these samples in various tasks (Zeng *et al.* 2021, Misek *et al.* 2022, Zhao *et al.* 2022). In our current study, we compared 369 HCC case samples against all samples from healthy controls (Fig. 1D, Supplementary Tables S1 and S2).

#### 2.1.2 Surface protein database

The COMPARTMENTS (Binder *et al.* 2014) database provides protein subcellular localization evidence built by integrating multiple sources like experimental datasets, sequence-based predictions, and manual literature curation, or by automated text-mining. Subcellular compartment terms were mapped to the cellular component subset of the Gene Ontology. Data from disparate sources were standardized by assigning confidence scores for all protein-compartment associations. We selected only 3806 surface protein-coding genes from this database based on confidence score >4 (Supplementary Table S3).

#### 2.1.3 Single-cell RNA-seq data

For the single-cell data of the case samples, we used 70 000 cells from the GEO dataset GSE149614 (Lu *et al.* 2022) which were collected from 10 HCC patients. For the control dataset, we used 10 372 cells from the liver single-cell atlas (Aizarani *et al.* 2019). For healthy vital organs control samples, we merged 10 360 cells from the lung (Vieira Braga *et al.* 2019), 8710 cells from the kidney nephrectomy (Ferreira *et al.* 2021), 6134 cells from the brain cortex (Habib *et al.* 2017), and 3785 cells from the left atrium and ventricle of a normal heart (Wang *et al.* 2020). We performed quality control (QC), normalization, and batch correction of the scRNA-seq data using Seurat (Satija *et al.* 2015) and Harmony (Korsunsky *et al.* 2019). We further used Seurat to process and normalize the data and then filtered out cells that expressed fewer than 200 genes or those with mitochondrial genes exceeding 5%. The remaining datasets underwent log-normalization with a 10k scale factor. The top 2000 variable features were selected for cell type labeling. The SingleR R package (Aran *et al.* 2019) which includes the human primary cell atlas dataset (Regev *et al.* 2017) was used as a reference to annotate cell types. The human cell atlas contains the expression of cells covering over 40 cell types.

#### 2.1.4 Spatial transcriptome data

Spatial transcriptomics (ST) is an emerging technology for profiling gene expression at the spatial resolution. We downloaded the HCC spatial transcriptome data from http://lifeome.net/supp/livercancer-st/data.htm (Wu *et al.* 2021). The ST tissue samples were processed using the SpaceRanger (10X Genomics) by the original authors.

### 2.2 Data analysis

#### 2.2.1 Identification of bsAbs pairs based on surface marker expression in bulk RNA-seq

High sensitivity and high specificity are two essential attributes of a candidate marker. High sensitivity indicates high expression rates in malignant tumor cells, while high specificity means that a great proportion of non-malignant cells do not express the marker. A qualified marker should be highly expressed in malignant cells and also should have low or zero expression in non-malignant cells in the same tissue. Moreover, it should exhibit low or no expression in vital organs such as the brain, liver, lung, kidney, and heart to minimize therapy-related toxicity.

We aim to identify bsAbs pairs not only based on their higher expression in case samples but also on how they maximally differentiate between case versus control samples. The pairwise combination of 3806 genes from the COMPARTMENT database has yielded over seven million distinct bsAbs pairs.

We used the following formula to identify promising bsAbs pairs:

$$\mathrm{pair}\;\mathrm{score}=\frac{1}{1+{\left(\frac{1-k}{1+k}\right)}^{2}}*\sqrt{{{(x}_{2}-x_{1})}^{2}{{(y}_{2}-y_{1})}^{2}}*\left(\mathrm{mean}\left(\frac{b_{i}-a_{i}}{\max\left\{b_{i},a_{i}\right\}\;}\right)+1\right)$$

where:

- *k* is the slope of the linear regression for case and control clusters,
-
$\frac{1}{1+{\left(\frac{1-k}{1+k}\right)}^{2}}$ is the cosine between the linear regression line and the *x*-axis,
- (*x*_1_, *y*_1_) are the coordinates of the medoid for the control cluster ‘*a*’,
- (*x*_2_, *y*_2_) are the coordinates of the medoid for the case cluster ‘*b*’,
-
$\frac{b_{i}-a_{i}}{\max\{b_{i},a_{i}\}}$ defines the silhouette of the case versus control cluster for each data point ‘*i*’.

The pair score solely assesses the potential of a marker pair to separate case and control samples as a bispecific antibody target, regardless of their expression levels in case or control samples. Therefore, to obtain the pairs for the case cluster, we also used the Boolean approach: both medoid coordinates for the case cluster should be greater than those for the control cluster, meaning both genes of the marker pair should exhibit high expression in the case cluster and low or negligible expression in the control cluster. Since the distribution of the scores was beta-like, to obtain *P*-values we used a permutation test with false discovery rate (FDR) correction. Next, to ensure higher specificity, we checked the expression of marker genes of significant bsAbs pairs in OCTAD reference normal vital organ samples (Supplementary Table S2).

To compute the sensitivity and specificity of candidate bsAbs pairs, we used support vector machine (SVM) available in R package e1071 along with the ROCR R package. We performed two separate sensitivity and specificity comparisons between dual markers (bsAbs) and single markers. Since the whole bsAbs target identification task can be viewed as a 2-feature classification problem, we trained two distinct SVM models: one for the case versus adjacent normal tissue and another for case versus vital organs. We computed the mean delta between the sensitivity and specificity of both models.

#### 2.2.2 Verification of cell-specific co-expression of the bsAbs pairs using scRNA-seq data

To confirm the bsAbs targets identified through bulk RNA-seq, we utilized the single-cell RNA-seq data. As HCC tumor is a mixture of immune cells, stromal cells, and hepatocytes, with the latter including malignant and non-malignant hepatocytes, it is important to concentrate only on the malignant cells in the tumor while computing true co-expression percentage. The inclusion of non-malignant parenchymal cells in bulk tumor tissues could have led to the identification of false-positive markers. To distinguish malignant cells from non-malignant ones, we referenced healthy hepatocytes obtained from the human liver cell atlas dataset (Aizarani *et al.* 2019) and used the scCancer package (Guo *et al.* 2021) which utilizes inferCNV package (Broadinstitute/Infercnv 2016/2023, Visualizing Large-Scale Copy Number Variation in Single-Cell RNA-Seq Expression Data n.d.) for malignancy prediction. After selecting the malignant cells from the scRNA-seq dataset, we validated the list of markers identified at the tissue level and chose those expressed in most malignant cells. Similar to the HCC dataset, we selected only primary hepatocytes from all other cells found in healthy liver samples. We annotated normal vital organ single cells according to a 3-level cell type classification system (Supplementary Table S5), similar to the methodology in the human cell atlas (Regev *et al.* 2017). The 3-level cell type classification system categorizes cells on hierarchical levels as major cell types (e.g. endothelial cells, epithelial cells, immune cells, neuronal cells, stromal cells, and tumor cells), subpopulations (e.g. liver epithelium and lung epithelium for epithelial cells), and individual cell states (levels of activation or specialization, such as B-cells, NK cells, plasma cells, and T-cells). Whenever possible, we used published classification results; in other cases, we used the scPred-based supervised classifier (Alquicira-Hernandez *et al.* 2019).

The co-expression of prominent markers was checked across individual single cells. To do this, we binarized the expression of every marker in each cell. An expression was marked as 1 if the expression surpassed a threshold (indicating presence) else 0 (indicating absence). This led to the creation of a Boolean matrix [*m* × *n*], where ‘*m*’ represents cells and ‘*n*’ denotes genes. Consequently, for any given gene pair in a specific cell, there are three potential outcomes: 0 (neither of the genes is expressed in the cell), 1 (one of the genes is expressed in the cell), and 2 (both genes are expressed in the cell). The significance of co-expression was decided based on *P*-values derived from the chi-square test with the FDR correction.

#### 2.2.3 Visualization of co-expression pattern using the spatial transcriptomic dataset

To further validate the co-expression of the top bsAbs target pair, we used the filtered ST data from the adjacent normal (control), tumor, and leading edge (the area between the tumor and the adjacent normal). We used the R package STUtility (Ludvig 2019, Bergenstråhle *et al.* 2020) to import all the data followed by integration of the expression data from different sections of each patient using harmony (Korsunsky *et al.* 2019). The Seurat package (Stuart *et al.* 2019) was used to perform downstream analysis and visualization.

## 3 Results

### 3.1 Selection of bsAbs target pairs based on surface marker expression in bulk RNA-seq

A recent study proposed bi-specific antibodies and tri-specific antibodies (recognizing two and three antigens) using large bulk RNA-seq databases *via* Boolean logic (Dannenfelser *et al.* 2020). The idea was to identify the markers that separate tumor samples from healthy samples with maximum heterogeneity. However, this study did not account for how good marker candidates are co-expressed. In the best-case scenario, clusters of case and control should be located in the opposite parts of the co-expression plot, and such location can be defined by the distance between clusters, and the slope of the linear regression for the cloud of samples and was determined by the ‘AND’ and ‘NOT’ logic. Specifically, it was based on the localization of the case cluster in the upper-right corner, where every antigen in the pair would have high expression, exemplified by (A AND B), while pairs with other case placements like (A NOT B), (NOT A AND B), or (NOT A NOT B) were disregarded. Nevertheless, it is possible to identify potential bispecific antigens using more robust methods, coupled with a conventional approach for the quantification of sensitivity and specificity. Our algorithm prioritizes gene pairs with maximum differences between case versus control and case versus healthy vital organs. Initially, we screened seven million gene pairs generated from pairwise combinations of 3806 unique genes from the COMPARTMENT database using our proposed approach (detailed in Section 2) to assess their ability to differentiate between case (HCC samples) and control (adjacent normal liver samples and vital organs). This screening identified over 300 pairs of genes significant in both score threshold and Boolean logic: both antigens showed greater expression in the case cluster (A & B), while the control cluster displayed double negative expression patterns (NOT A NOT B) (S4).

**Figure 2.**
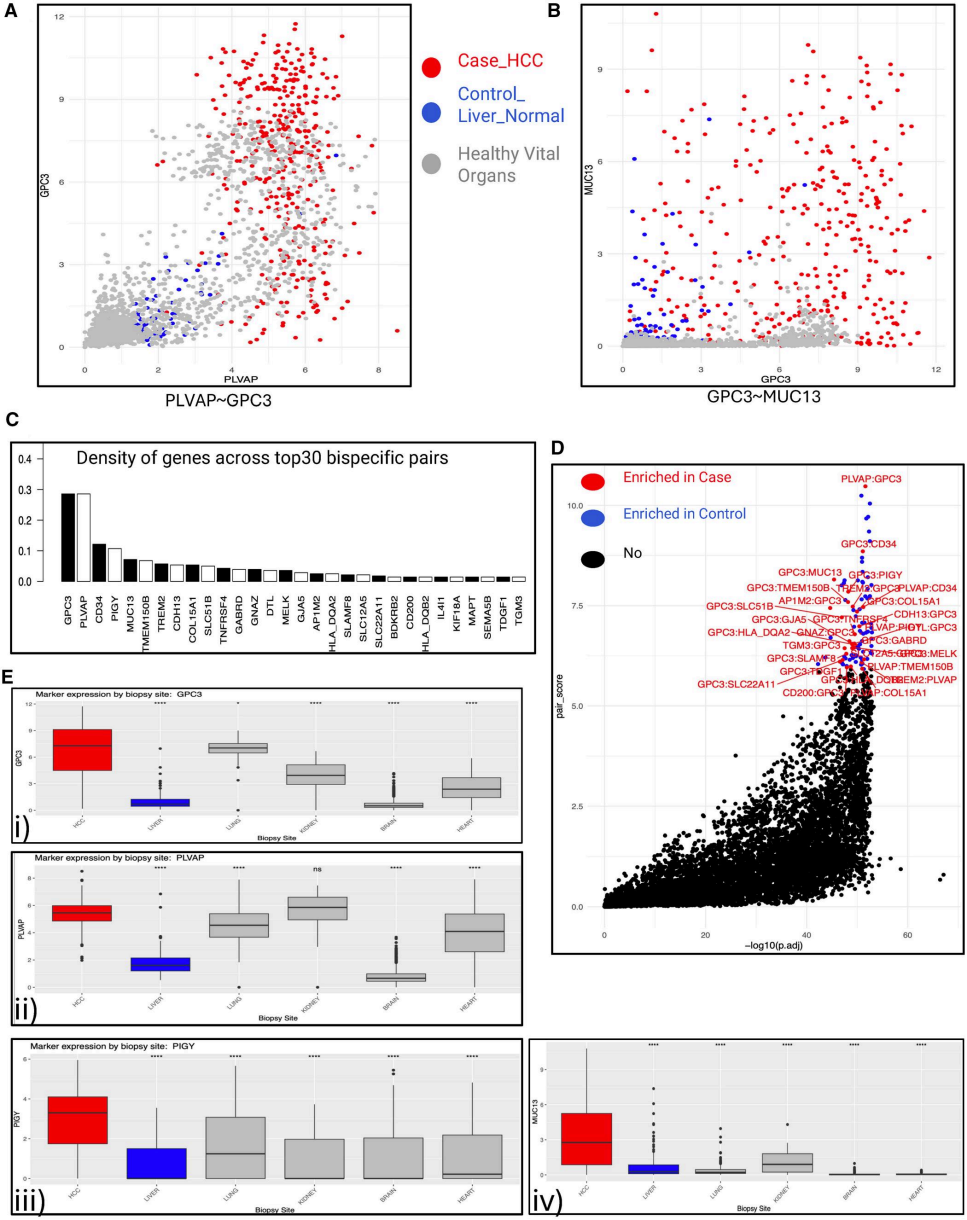
Selection of top bsAbs target pairs using OCTAD bulk RNA-seq. (A, B) Expression distribution of potential marker pairs GPC3–PLVAP (A) and GPC–MUC13 (B) in tumors (red), adjacent normal tissues (blue), and normal vital organs (grey). (C) Frequencies of the most common potential markers for the bsABs candidates. (D) Score and significance distribution of the proposed bsAbs pairs based on the pair score and permutation-obtained significance scores. (E(i–iv)) Expression of the four topmost frequent genes of the bsAbs pairs in HCC, normal liver, and normal vital organs.

Figure 2A–D showcases all the output plots generated by our package. Specifically, Fig. 2D illustrates all pair combinations, with red indicating enrichment in the case cluster. Figure 2A and B exemplifiers potentially avoiding (PLVAP–GPC3) and promising pair (GPC3–MUC13), respectively, with interpretations provided later.

Frequency analysis of genes in bispecific target pairs revealed a consistent presence of GPC3 across most pairs, followed by PLVAP, CD4, PIGY, and MUC13 (Fig. 2C). Considering the prevalence of GPC3 as a biomarker and therapeutic target in HCC (Koksal *et al.* 2022, Wu *et al.* 2022), its presence in the top most candidate genes was anticipated, confirming the validity of our approach. However, to ensure marker specificity with minimal therapy-related toxicity, the expression of these frequent marker genes was compared in HCC samples versus normal liver and other vital organs (Fig. 2E).

For instance, although PLVAP–GPC3 showed optimal performance in HCC, PLVAP shows limited specificity, with comparable levels detected in the lung, kidney, and heart (Fig. 2E-ii), making it an unfavorable pair, as illustrated in Fig. 2A. Similarly, in the CD34–GPC3 pair, CD34 specificity for hematopoietic stem and progenitor cells suggests potential identification of false-positive pairs using only bulk RNA-seq (Supplementary Table S6). Notably, among all genes in predicted pairs, only MUC13 showed specific expression in HCC, with little to no expression in vital organs (Fig. 2B and E-iv).

**Figure 3.**
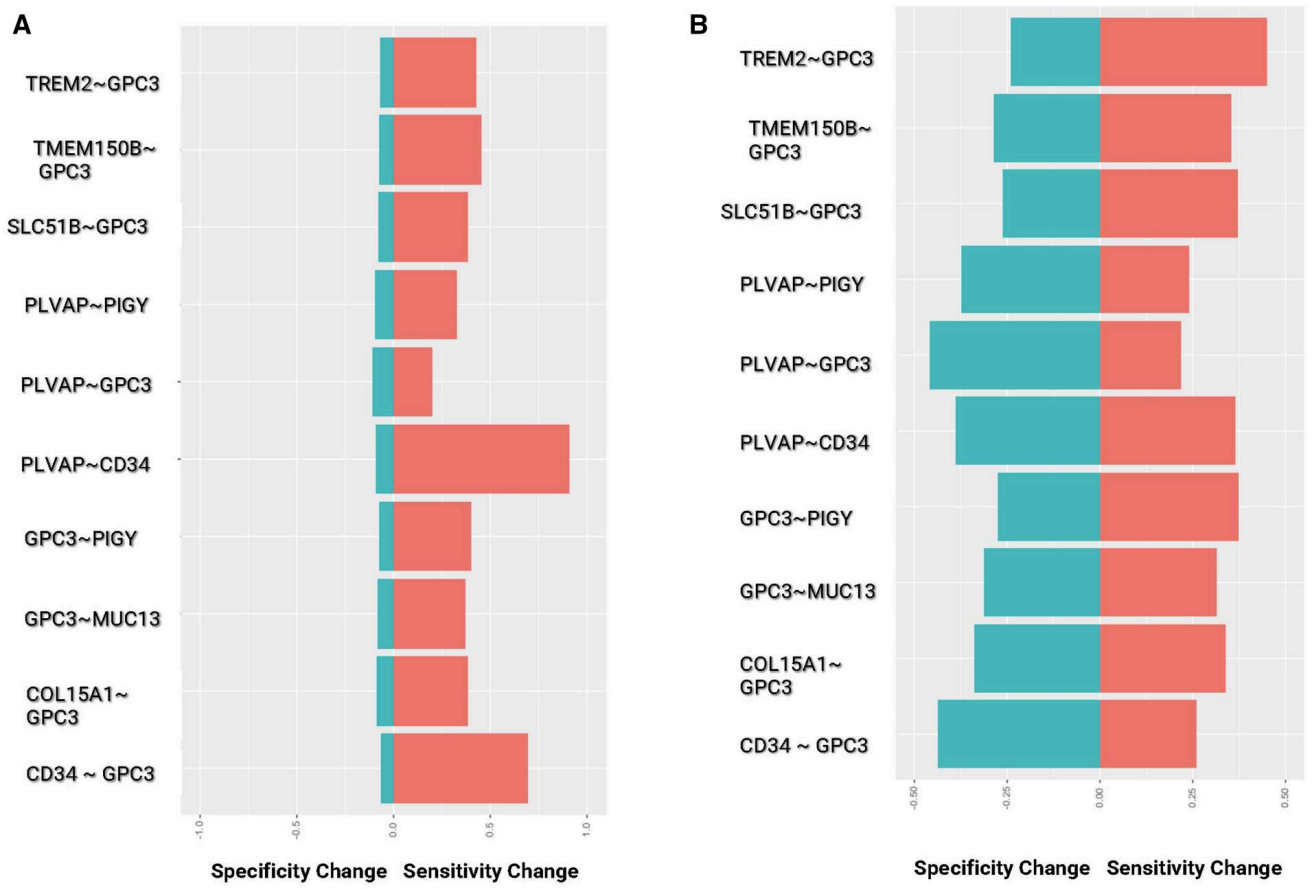
Sensitivity and specificity analysis between dual targeting and single-agent targeting from bulk RNA-seq data. Increase of sensitivity and specificity between bsAbs and single agents while using adjacent normal tissues (A) or vital organs (B) as the control.

The ability of top candidate marker pairs to separate between tumors versus adjacent normal tissue and vital organs was further assessed by comparing the sensitivity and specificity of SVM models. The mean delta between the sensitivity and specificity for bsAbs pairs and individual markers in the pairs is shown in Fig. 3. While we noted a minor increase in specificity for the tumor versus adjacent tissue comparison, a considerable boost in sensitivity was observed for the same comparison (Fig. 3A, Supplementary Table S7). Moreover, both sensitivity and specificity presented a marked improvement in comparison between tumors and vital organs (Fig. 3B, Supplementary Table S8). This emphasizes the superiority of dual targeting over single-agent targeting.

### 3.2 scRNA-seq quantifies cell-specific co-expression of the bsAbs

**Figure 4.**
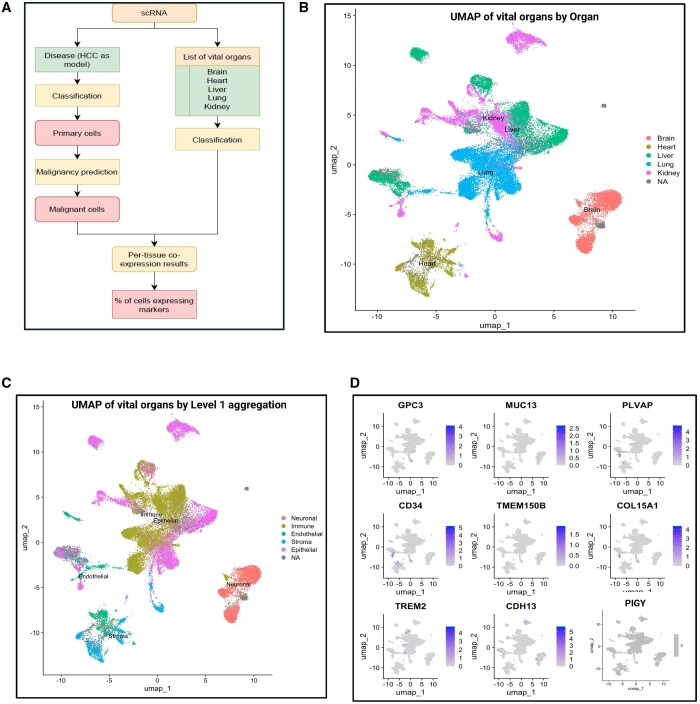
(A) Principal scheme of the scRNA-seq-based bsAbs verification. (B) UMAP of the vital organ scRNA-seq data used for the verification of cytotoxicity results. (C) UMAP of the level 1 aggregation to define endothelial, epithelial, immune, neuronal, and stromal cells in the vital organs database. (D) Expression of the top nine most frequent marker genes across vital organs.

Our findings prompted us to leverage the scRNA-seq for the validation of bsAbs derived from bulk RNA-seq, aiming for the discovery of bsAbs with high tissue specificity and minimal non-specific cytotoxicity. To check the expression of promising marker genes in the scRNA-seq dataset, we followed a similar approach as in bulk RNA-seq (Fig. 4A). Figure 4B and C visualizes the UMAP of integrated vital organ datasets according to organs and main cell types, respectively. To assess non-specific cytotoxicity, we visualized the expression level of selected markers on cell surfaces of the vital organs (Fig. 4D). Even if some selected pairs showed expression in the vital organs, they might remain viable if not expressed in the parenchymal cells of those vital organs.

**Figure 5.**
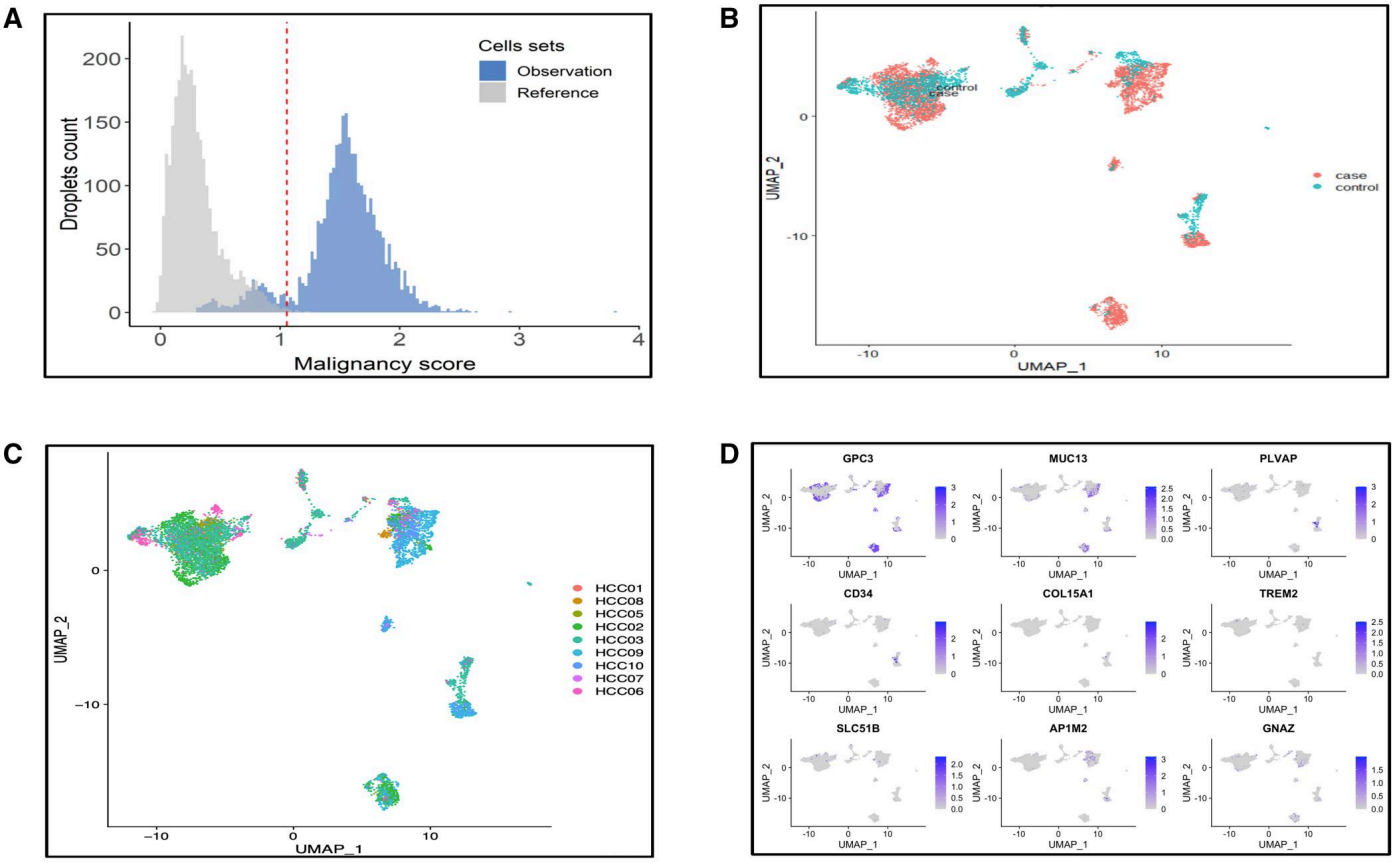
Tumor cell classification and cell malignancy inference from the HCC scRNA-seq dataset. (A) Malignance score distribution of the reference hepatocytes versus cells from one HCC cell line to leverage only malignant hepatocytes from the tumor data for downstream analysis. (B) Distribution of the malignant (case) and non-malignant (control) hepatocytes from the HCC scRNA-seq dataset. (C) UMAP showing heterogeneity among patients from the HCC scRNA-seq dataset. (D) Expression of the most frequent marker genes in the HCC malignant hepatocytes.

For the identification of highly sensitive pairs, it is important to concentrate only on the malignant hepatocytes in HCC scRNA-seq data while computing the true co-expression percentage. We classified cells and extracted only hepatocytes, and identified malignant cells among them. By establishing a threshold derived by comparing Huh-7 cells with normal reference hepatocytes (Fig. 5A), out of the 7140 hepatocytes identified, 5271 were malignant, and 1869 were non-malignant (Fig. 5B). Heterogeneity within the patient was observed, with the malignant hepatocytes clustering into five subpopulations, each displaying distinct transcriptional features (Fig. 5C). The expression level of selected markers varied across filtered hepatocytes (Fig. 5D).

**Figure 6.**
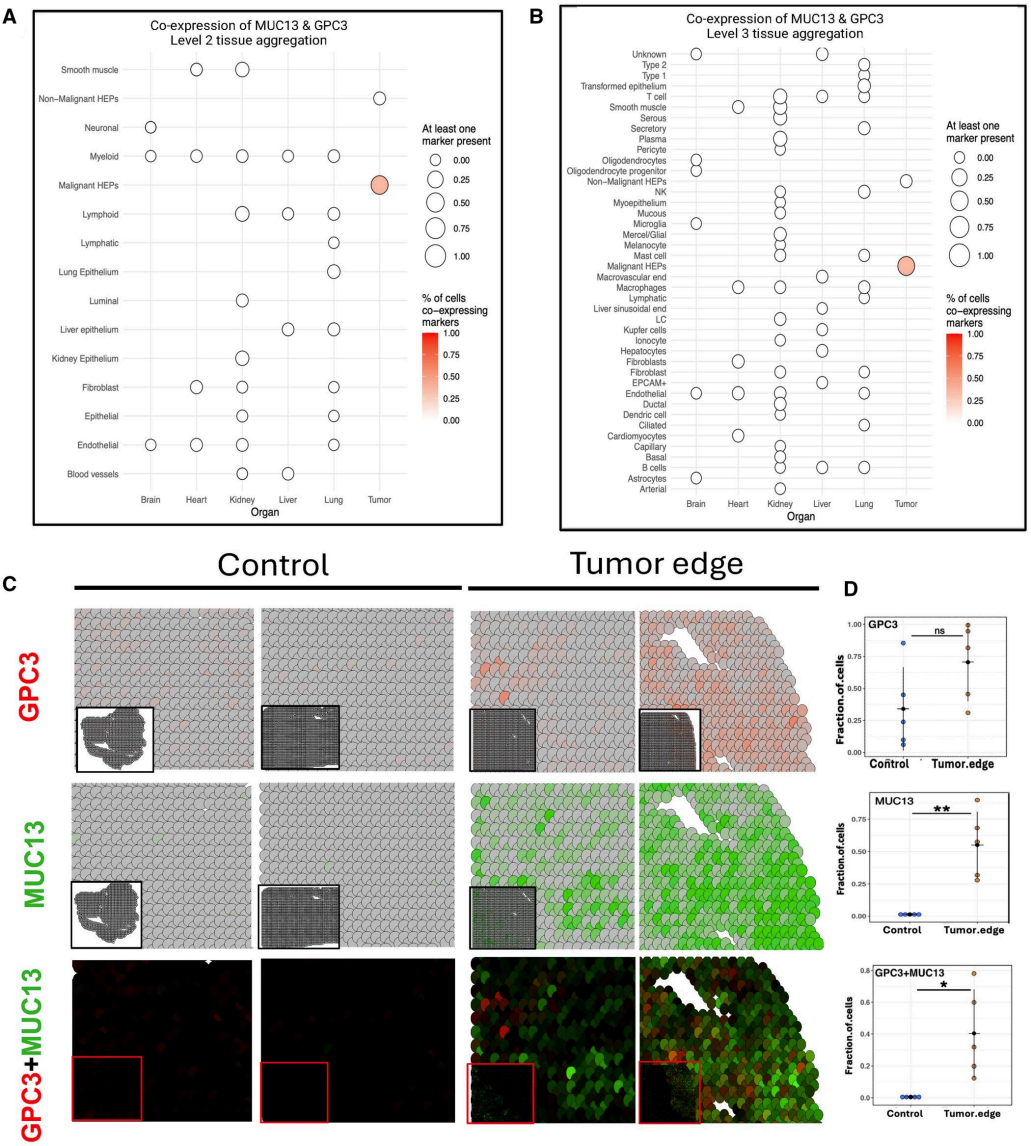
Single-cell and spatial transcriptome analysis both confirm the potential of the GPC3–MUC13 marker pair as \ HCC bsAbs targets. (A) Single-cell co-expression analysis of the pair on the level II aggregation. (B) Single-cell co-expression analysis of the pair in vital organs and malignant hepatic cells on the level III aggregation. (C) Expression and co-expression of GPC3 and MUC13 in two control samples and two tumor spatial transcriptome. (D) Percentage of cells expressing and co-expressing GPC3 and MUC13 in spatial datasets.

The last phase aimed to verify the tissue specificity of the favorable markers at single-cell levels. We first transformed the markers into binary matrices, assigning 1 to a cell if the count surpassed a threshold and 0 otherwise. This binary transformation allowed us to compute the co-expression of the markers across every identified cell type. In HCC, the most promising marker pair turned out to be GPC3–MUC13. Both markers of this pair were co-expressed in 30% of malignant cells and were barely detected in vital organs. This profile suggests the potential of this target pair as a promising candidate for HCC therapy using bsAbs (Fig. 6A and B).

### 3.3 Validation of specificity and sensitivity of topmost bsAbs pair using spatial transcriptomic data

The spatial transcriptomic analysis unveiled a distinctive pattern in the tumor microenvironment, where both GPC3 and MUC13 were highly expressed in tumor samples contrasting sharply with their complete absence in the control sample (Fig. 6C and Supplementary Fig. S1). GPC3 and MUC13 were co-expressed in approximately 40% of tumor cells (Fig. 6D and Supplementary Fig. S3).

## 4 Discussion and conclusion

Our newly developed computational pipeline underscores the strength of an integrated approach in the unbiased survey of bsAbs candidates for a cancer. By fuzing the complementary advantages of both bulk and single-cell RNA sequencing, we can accentuate their positive attributes while simultaneously offsetting the limitations inherent when each is employed individually. Examining the spatial distribution of these markers within tumors proved valuable while assessing their potential in bsAbs development. One salient insight from our HCC case study was the discernible value added by scRNA-seq in curtailing the chances of discovering false-positive pairs derived from bulk RNA-seq. The visualization of the top pair GPC3–MUC13 *in situ* further provided evidence for their co-expression in tumor cells, suggesting their potential as a therapeutic target pair.

Additionally, two more bulk datasets, namely GSE77314 (Liu *et al.* 2016) and GSE124535 (Zeng *et al.* 2020), were analysed. GSE77314 comprised sequencing data from 50 paired samples of HCC and adjacent normal tissues, while GSE124535 included 35 paired samples. Consistent with our findings, these datasets further demonstrate elevated expression levels of GPC3 and MUC13 in HCC compared to control samples (Supplementary Fig. S1A and B).

Two additional single-cell datasets GSE151530 (Ma *et al.* 2021) and GSE189903 (Ma *et al.* 2022) from Single-cell Atlas in Liver Cancer (scAtlasLC) (Single-cell Atlas in Liver Cancer n.d.) revealed the presence of malignant cells expressing GPC3 and MUC13. However, the co-expression was low to negligible due to the heterogeneous nature of malignant cells across different patients. One potential bispecific antibody can target either GPC3+ or MUC13+ cells for increasing the coverage of cancer cells.

The therapeutic potential of the markers identified in our study for HCC is supported by existing literature. Previous studies have identified GPC3 (Zheng *et al.* 2022) and MUC13 (Dai *et al.* 2018) as individual targets for HCC treatment. GPC3 specific antibody drug conjugates (ADC) targeting HCC have also been developed (Fu *et al.* 2019).

As per our current knowledge, none of the bsAbs with dual-targeting capabilities have been approved for HCC treatment. The majority of clinical bsAbs are typically engineered as T-cell immune engagers (Yu *et al.* 2020), which harness the immune system to target malignant cells. In contrast, our approach focuses on identifying cell surface antigens that are specifically expressed on malignant cells, which distinguishes it from the more commonly observed T-cell engagement strategy. Further, some bsAbs rely on targets that are functionally related to survival or tumor growth promotion, while many others only require the expression of these targets on malignant cells for antibody recognition. Our approach aligns with this, emphasizing target expression rather than functional associations.

While our current study focuses on HCC, our pipeline’s flexibility and adaptability enable swift recalibration for identifying bsAbs suitable for other cancers. Considering the potential discordance between RNA expression and corresponding protein expression due to various factors including post-transcriptional modifications and protein stability, the promising marker pairs identified through our computational pipeline must undergo subsequent benchmark validation. Techniques such as immunostaining or other protein quantification methods like MIBI-TOF, Codex, spatial CITE-seq would be instrumental in this confirmatory phase.

## Supplementary Material

vbae096_Supplementary_Data

## Data Availability

All bulk data used in the article are available online: OCTAD database. (https://github.com/Bin-Chen-Lab/octad). Also, a standalone R package for bsAbs testing in bulk data is available *via* GitHub https://github.com/Bin-Chen-Lab/bsAbsFinder. Example_Code folder on GitHub includes related scripts. In addition, we have provided code to programmatically download single-cell data from the TISCH (Sun *et al.* 2021, Han *et al.* 2023) database for a variety of cancers. Links and code for single-cell data analysis are also provided in the GitHub.
