# Supplementary figures and images for: Computational discovery of co-expressed antigens as dual targeting candidates for cancer therapy through bulk, single-cell, and spatial transcriptomics
Source: Bioinform Adv. 2024 Jun 20;4(1):vbae096. doi: 10.1093/bioadv/vbae096 (PMC11770384; doi:10.1093/bioadv/vbae096)

A)

GSE77314 - GPC3

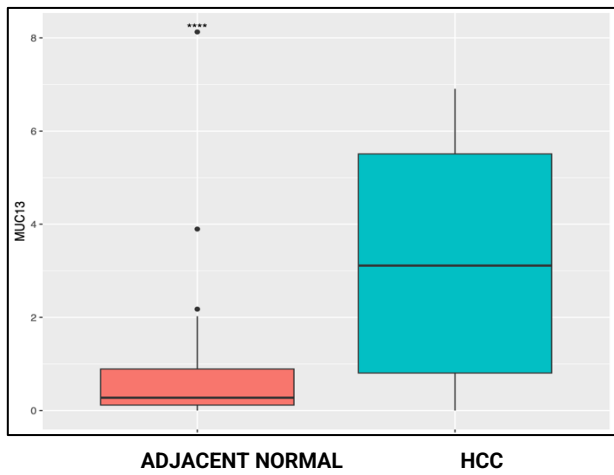

B)

GSE124535 - GPC3

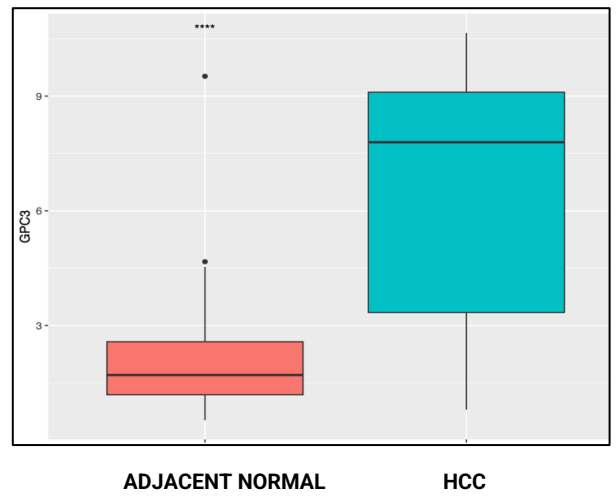

GSE77314 - MUC13

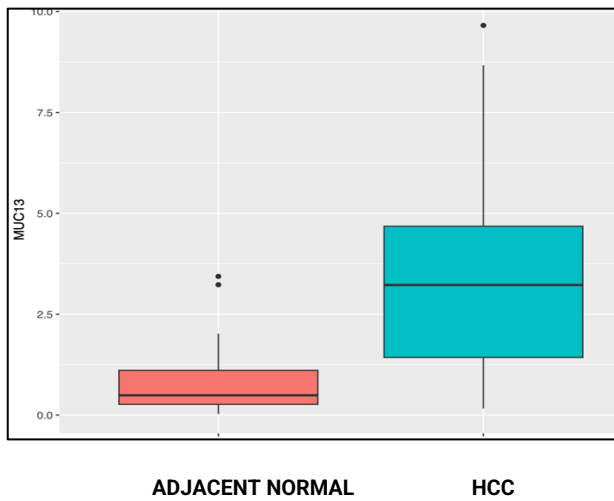

GSE124535 - MUC13

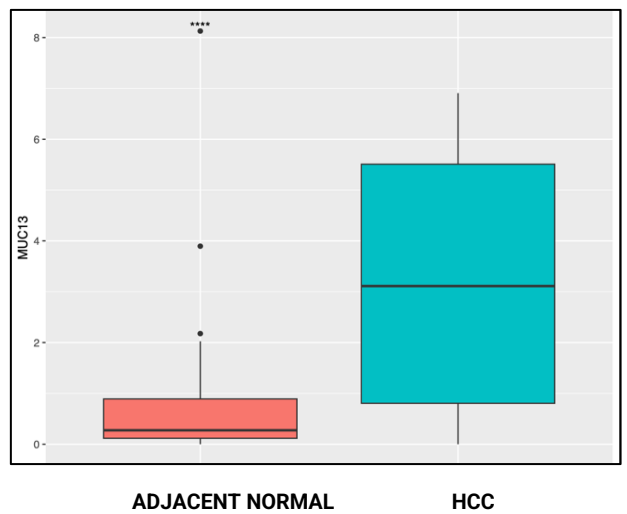

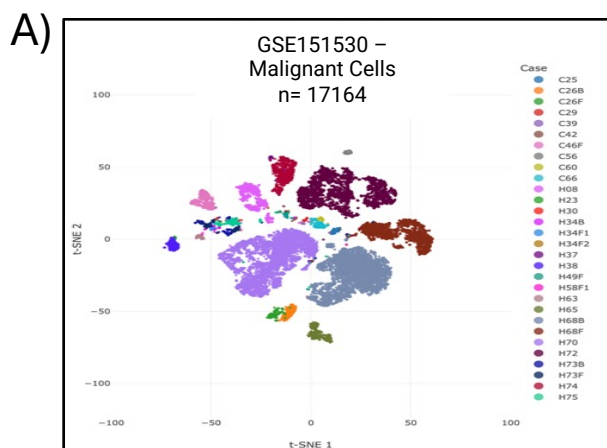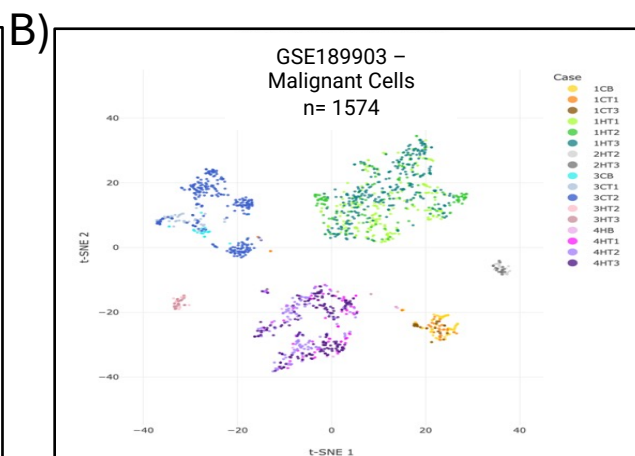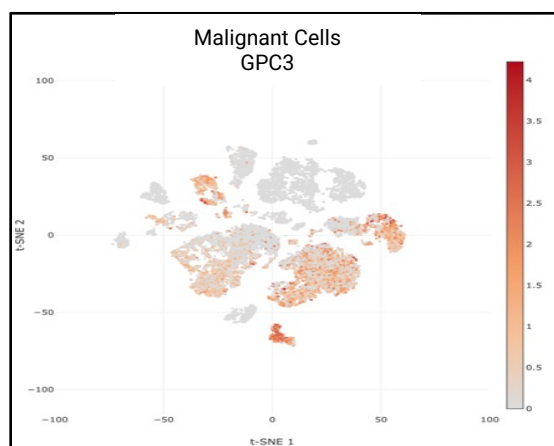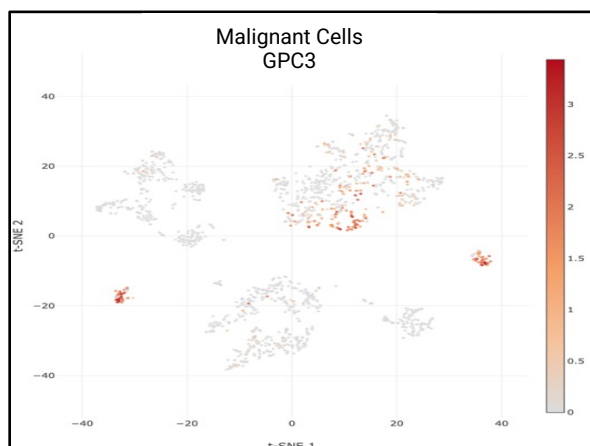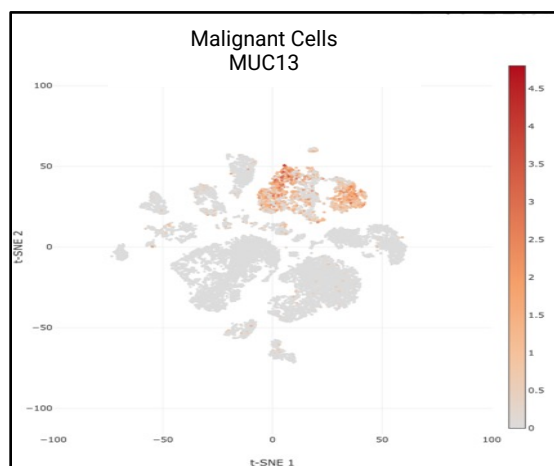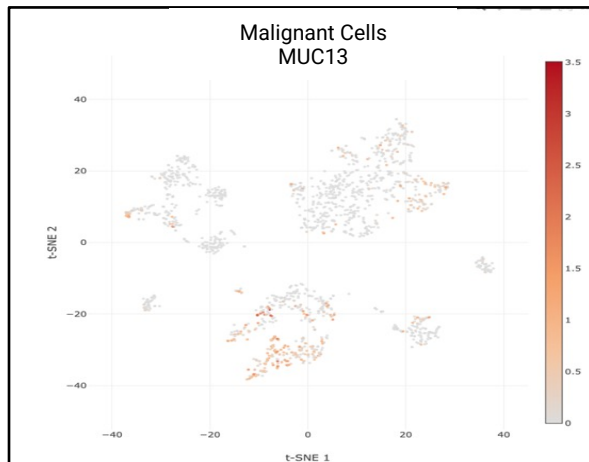

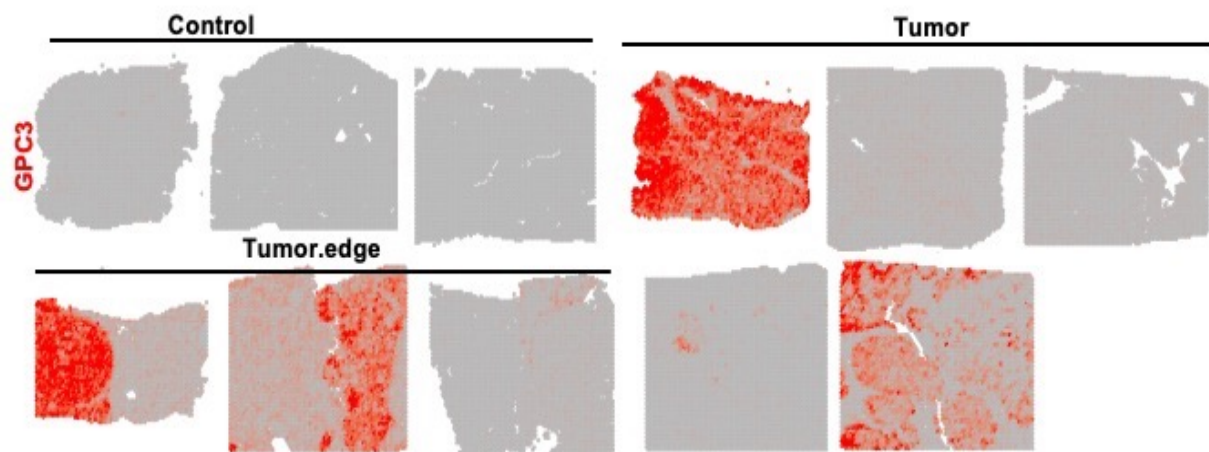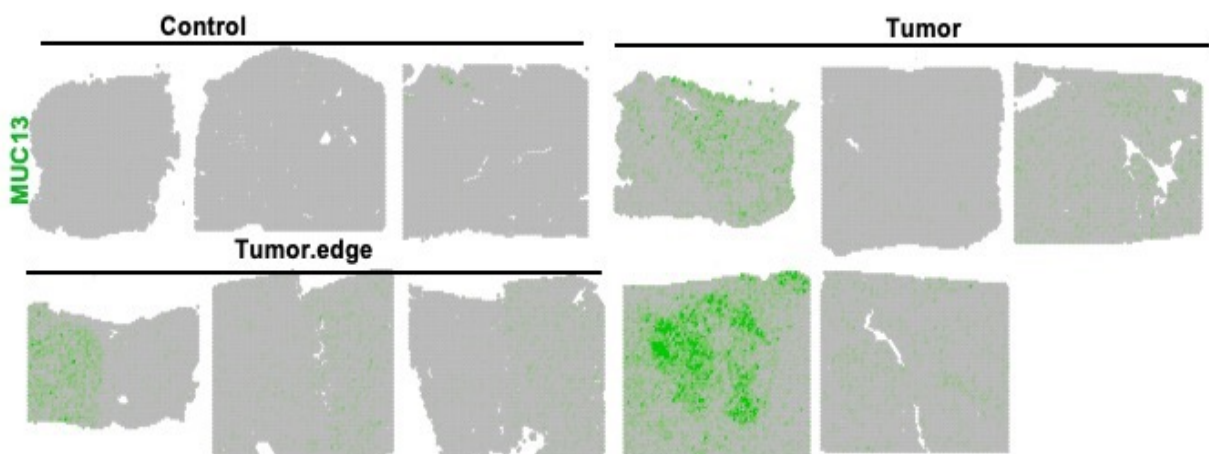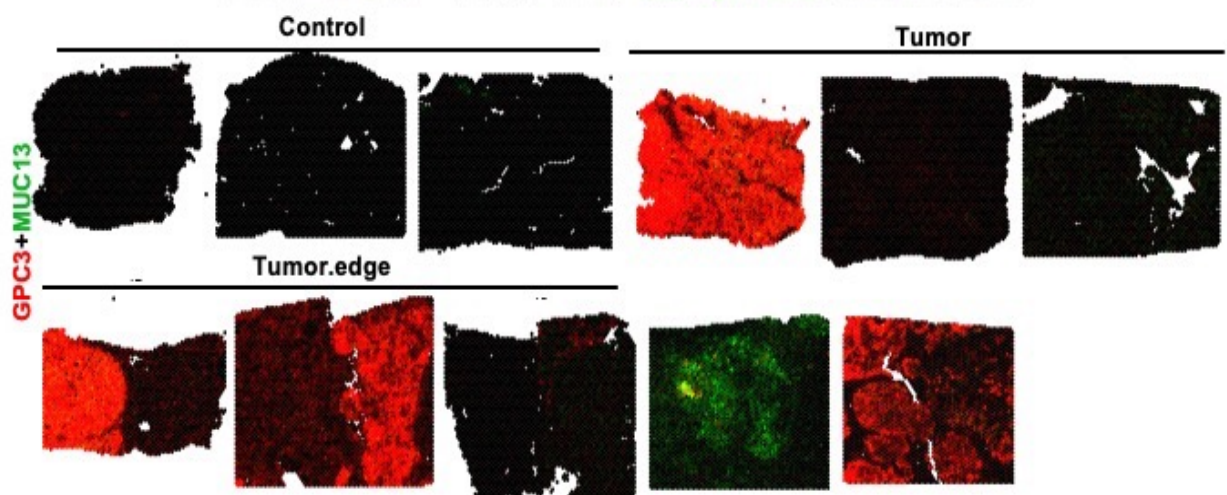

Supplement: vbae096_Supplementary_Data [file vbae096_supplementary_data.zip › BSABS_FIGURES_SUPP.pdf]
